# Platycodin D, a natural component of *Platycodon grandiflorum*, prevents both lysosome- and TMPRSS2-driven SARS-CoV-2 infection by hindering membrane fusion

**DOI:** 10.1038/s12276-021-00624-9

**Published:** 2021-05-25

**Authors:** Tai Young Kim, Sangeun Jeon, Youngho Jang, Lizaveta Gotina, Joungha Won, Yeon Ha Ju, Sunpil Kim, Minwoo Wendy Jang, Woojin Won, Mingu Gordon Park, Ae Nim Pae, Sunkyu Han, Seungtaek Kim, C. Justin Lee

**Affiliations:** 1 Center for Cognition and Sociality, Cognitive Glioscience Group, Institute for Basic Science, Daejeon, 34126 Republic of Korea; 2 Zoonotic Virus Laboratory, Institut Pasteur Korea, Seongnam, Republic of Korea; 3 Department of Chemistry, Korea Advanced Institute of Science and Technology (KAIST), Daejeon, 34141 Republic of Korea; 4 Convergence Research Center for Diagnosis, Treatment and Care System of Dementia, Korea Institute of Science and Technology, Hwarangno 14-gil 5, Seongbuk-gu, Seoul, 02792 Republic of Korea; 5 Division of Bio-Medical Science & Technology, KIST School, Korea University of Science and Technology, Daejeon, Republic of Korea; 6 Department of Biological Sciences, Korea Advanced Institute of Science and Technology (KAIST), Daejeon, 34141 Republic of Korea; 7 IBS School, University of Science and Technology, Daejeon, Republic of Korea; 8 Neuroscience Program, University of Science and Technology, Daejeon, Republic of Korea; 9 KU-KIST Graduate School of Converging Science and Technology, Korea University, Seoul, 02841 Republic of Korea

**Keywords:** Viral infection, Lipid signalling

## Abstract

An ongoing pandemic of coronavirus disease 2019 (COVID-19) is now the greatest threat to global public health. Herbal medicines and their derived natural products have drawn much attention in the treatment of COVID-19, but the detailed mechanisms by which natural products inhibit SARS-CoV-2 have not been elucidated. Here, we show that platycodin D (PD), a triterpenoid saponin abundant in *Platycodon grandiflorum* (PG), a dietary and medicinal herb commonly used in East Asia, effectively blocks the two main SARS-CoV-2 infection routes via lysosome- and transmembrane protease serine 2 (TMPRSS2)-driven entry. Mechanistically, PD prevents host entry of SARS-CoV-2 by redistributing membrane cholesterol to prevent membrane fusion, which can be reinstated by treatment with a PD-encapsulating agent. Furthermore, the inhibitory effects of PD are recapitulated by the pharmacological inhibition or gene silencing of *NPC1*, which is mutated in patients with Niemann–Pick type C (NPC) displaying disrupted membrane cholesterol distribution. Finally, readily available local foods or herbal medicines containing PG root show similar inhibitory effects against SARS-CoV-2 infection. Our study proposes that PD is a potent natural product for preventing or treating COVID-19 and that briefly disrupting the distribution of membrane cholesterol is a potential novel therapeutic strategy for SARS-CoV-2 infection.

## COVID-19: Closing the cellular door on coronavirus

A molecule derived from a traditional Korean herbal medicine can impede entry of SARS-CoV-2 into cultured cells. The coronavirus responsible for COVID-19 can enter the cell via direct fusion with the plasma membrane, or after internalization into organelles called lysosomes. South Korean researchers led by Seungtaek Kim of the Institut Pasteur Korea, Seongnam, and C. Justin Lee at the Institute for Basic Science, Daejon, have identified a compound that can thwart both modes of entry. They treated cultured human cells with platycodin D (PD), extracted from a plant used throughout East Asia as a remedy for respiratory disease. The researchers showed that PD inhibits SARS-CoV-2 fusion with the plasma membrane while also blocking lysosomal release. This mechanism appears to be mediated by PD’s influence on membrane cholesterol content, suggesting a novel strategy for treating COVID-19.

## Introduction

SARS-CoV-2 expresses spike glycoprotein (S) on its surface and uses it to bind to the host receptor angiotensin-converting enzyme 2 (ACE2)^1,2^. SARS-CoV-2 is known to enter host cells during infection via two pathways: (1) the endocytic pathway, followed by cathepsin B/L-mediated cleavage of the S protein in lysosomes and (2) direct fusion of the virus envelope with the host plasma membrane after TMPRSS2-mediated cleavage of the S protein^2,3^. The cleavage of the S protein by cathepsin B/L, TMPRSS2 or both is the critical step to release viral RNA into the cytosol of host cells. Therefore, the abundances and availabilities of the two host proteases cathepsin B/L and TMPRSS2 and the host receptor ACE2 are the most critical factors determining host susceptibility to COVID-19. For example, chloroquine, a well-known anti-malaria drug, was initially suggested to have potent anti-SARS-CoV-2 activity due to its ability to block lysosomal cathepsin-dependent virus entry by elevating the lysosomal pH^4,5^. Unfortunately, most clinical trials with chloroquine and hydroxychloroquine failed to show beneficial effects in COVID-19 patients^6^. The failure of chloroquine was attributed to its inability to block TMPRSS2-mediated SARS-CoV-2 entry^7^. Subsequently, TMPRSS2 inhibitors such as camostat and nafamostat emerged as next-generation blockers of SARS-CoV-2 entry. However, they also fell short due to their inability to block SARS-CoV-2 entry into certain cell types that express only ACE2 without TMPRSS2^8,9^. Therefore, the discovery and development of drugs that block both lysosome- and TMPRSS2-driven SARS-CoV-2 entry are desperately needed.

**Fig. 1:**
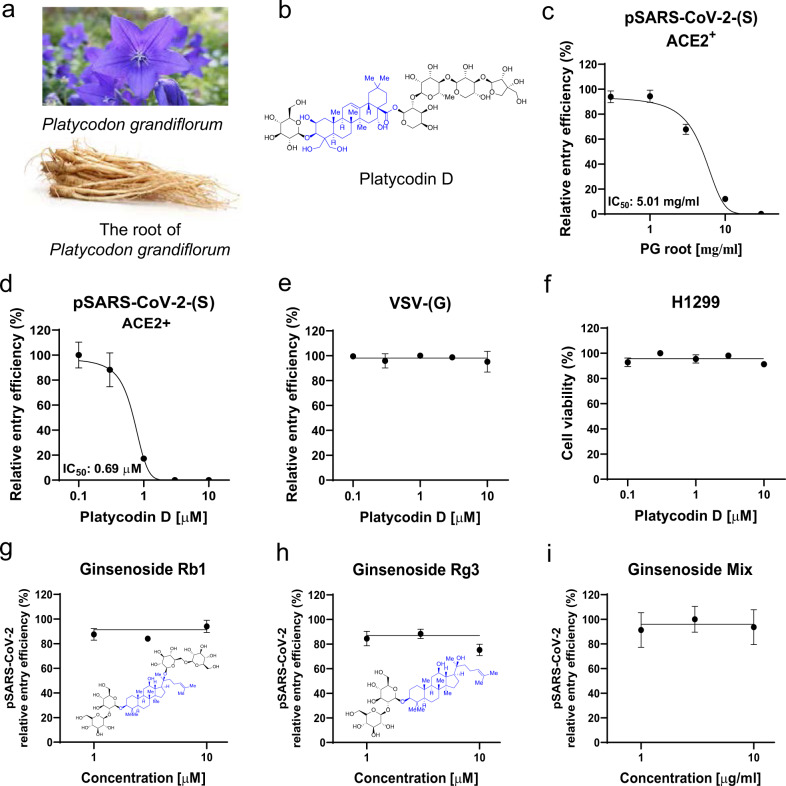
PD, a saponin present only in *Platycodon grandiflorum*, blocks SARS-CoV-2 entry into human cells. **a** *Platycodon grandiflorum* (balloon flower) and the root of *Platycodon grandiflorum*. **b** Chemical structure of PD, with triterpenoid saponin shown in blue. **c–e** ACE2^+^ H1299 cells were pretreated for 1 h with the indicated concentrations of water extract of PG root (**c**) or PD (**d**, **e**) and transduced with pSARS-CoV-2-(S) (**c**, **d**) or VSV-(G) (**e**). After culture for 24 h, the transduction efficiency was quantified by measuring the activity of firefly luciferase in the cell lysates. **f** Cytotoxicity was determined using the WST-8 cell viability assay. **g–i** pSARS-CoV-2 entry assay with ginsenoside Rb1 (1, 3, 10 μM) (**g**), Rg3 (1, 3, 10 μM) (**h**), and the ginsenoside mixture (1, 3, 10 μg/ml) (**i**). The inset shows the chemical structures of Rb1 and Rg3 (**g**, **h**). The data were representative of three independent experiments with triplicate samples. The error bars indicate the SEM.

Herbal medicines and their derived natural products have drawn much attention in regards to COVID-19 treatment because they have been shown to possess antiviral activity against a broad range of pathogenic viruses, including influenza, HIV, SARS-CoV, and MERS-CoV^10^. To date, numerous natural products have been proposed to inhibit one of the essential components of SARS-CoV-2, including main protease (Mpro), RNA-dependent RNA polymerase (RdRp), ACE2, and TMPRSS2; however, most are based on in silico screening through molecular docking approaches^11–13^. The inhibitory activities have been verified for only a few of these natural products^14^, and the detailed molecular mechanisms have been elucidated for none. Among thousands of Korean traditional herbal medicines, we have focused on the root of *Platycodon grandiflorum* (PG) (Fig. 1a), which is described in Dongui Bogam, the most famous 17th century Korean medical textbook^15^. The Dongui Bogam reports that PG can be used to treat patients with disorders of the respiratory tract and lung, the major target sites of SARS-CoV-2^16^. PG continues to be widely used in East Asian countries, including Korea, China, and Japan, to treat several respiratory ailments, such as asthma, airway inflammation, and sore throats^17–19^. Several studies have demonstrated that the root of PG is enriched with platycodin D (PD) (Fig. 1b), a glycosylated triterpenoid saponin (colored in blue; Fig. 1b) and the major active natural component that mediates these biological activities^20,21^. Recently, PD has been reported to exhibit potent antiviral activity against type 2 porcine reproductive and respiratory syndrome virus (PRRSV) and hepatitis C virus (HCV)^22,23^. However, its inhibitory activity against coronaviruses and its mechanism of action have not been explored. In this study, we investigated whether PG and PD show anti-SARS-CoV-2 activity by blocking both lysosome- and TMPRSS2-driven SARS-CoV-2 entry.

## Results

### PD, a triterpenoid saponin present in *Platycodon grandiflorum*, has specific inhibitory activity against SARS-CoV-2 entry

To test for anti-SARS-CoV-2 activity, we first developed a SARS-CoV-2 pseudovirus (pSARS-CoV-2) that carried the full-length S protein of SARS-CoV-2 on HIV-based lentiviral particles and the luciferase gene as a reporter^24^ to mimic the S protein of the native SARS-CoV-2 virus and retain its ability to bind to host cell surface receptors for viral infection. We then examined whether PG and PD could prevent pSARS-CoV-2 entry into H1299 cells, a human lung cell line known to be susceptible to coronavirus infection^25^. A luciferase activity assay after infection with pSARS-CoV-2 revealed that virus entry into H1299 cells required overexpression of ACE2 (ACE2^+^) (Supplementary Information, Fig. S1). In ACE2^+^ cells, we found that 1 h of treatment with PG and PD effectively reduced pSARS-CoV-2 entry in a dose-dependent manner with half-maximal inhibitory concentrations (IC_50_) of 5.01 mg/ml (Fig. 1c) and 0.69 μM (Fig. 1d), respectively. In contrast, PD did not block the entry of the control lentiviral particles driven by the glycoprotein (G proteins) of the vesicular stomatitis virus (VSV) (Fig. 1e). PD showed no cytotoxic effect on H1299 cells at the tested concentrations (Fig. 1f). These results indicate that the inhibitory effect of PD on virus entry requires both the S protein of SARS-CoV-2 and ACE2 in host cells. To test the specificity of PD among other saponins, we compared it with ginsenosides, which are a group of saponins from *Panax ginseng*, also known as Korean ginseng. Ginsenosides are known to exhibit antiviral activity against multiple types of viruses, such as rhinovirus, influenza virus, HIV, hepatitis virus, and herpesvirus^26^. In the pSARS-CoV-2 entry assay, none of the ginsenosides we tested, including the Rb1, Rg3, and ginsenoside mixture, prevented pSARS-CoV-2 from entering ACE2^+^ cells (Fig. 1g–i), suggesting that PD, a triterpenoid saponin present only in *Platycodon grandiflorum*, possesses specific inhibitory activity against SARS-CoV-2 entry.

### Herbal medicine and foods containing PG inhibit both the lysosome- and TMPRSS2-mediated SARS-CoV-2 entry pathways through the action of PD

**Fig. 2:**
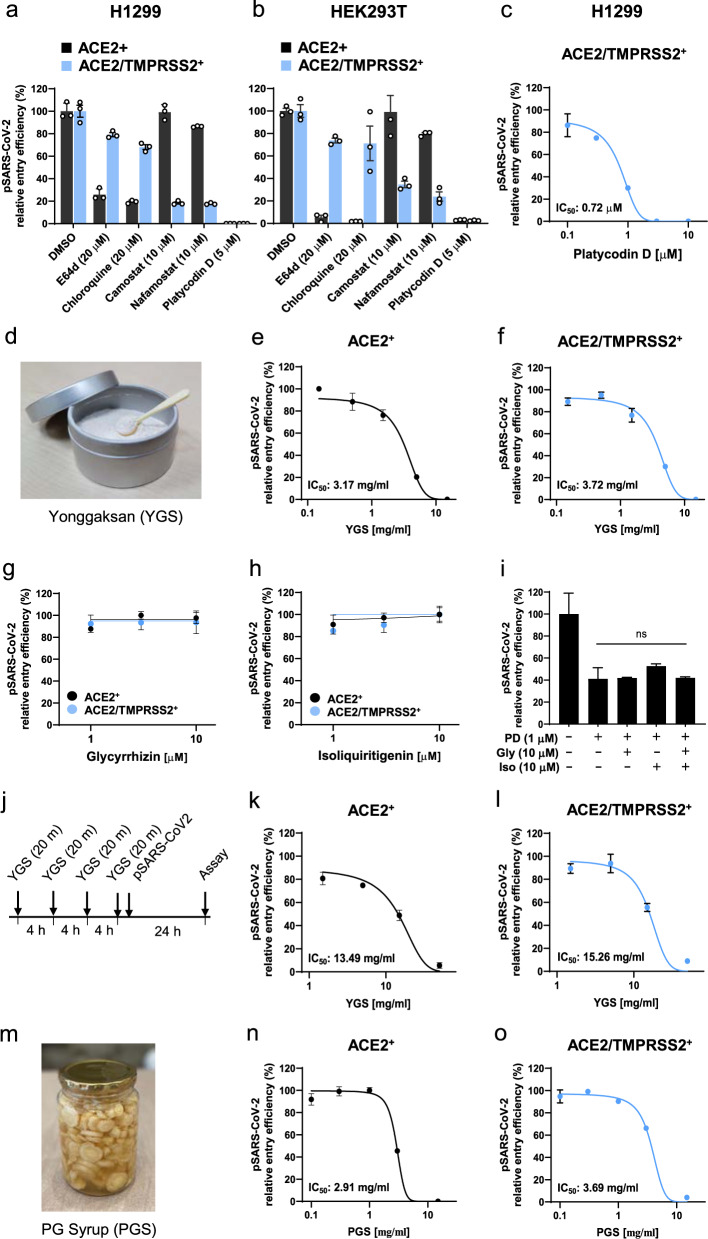
PD and PG root-containing herbal medicines and foods block two main SARS-CoV-2 entry pathways. **a**, **b** H1299 cells (**a**) and HEK293T cells (**b**) that expressed ACE2 alone (ACE2^+^) or in combination with TMPRSS2 (ACE2/TMPRSS2^+^) were pretreated with each drug for 1 h prior to the pSARS-CoV-2 entry assay. The lysosomal protease inhibitors E64d and chloroquine and the TMPRSS2 inhibitors camostat and nafamostat were used to verify the pSARS-CoV-2- entry pathways in these cells. **c** pSARS-CoV-2 entry assay with PD in ACE2/TMPRSS2^+^ cells. **d** Yonggaksan (YGS) is composed of a group of herbal powders, including PG root. **e**–**i** ACE2^+^ and ACE2/TMPRSS2^+^ cells were treated with serial threefold dilutions of the YGS stock solution (15 mg/ml) (**e**, **f**), with 1, 3, 10 μM glycyrrhizin and isoliquiritigenin (**g**, **h**), or with 1 μM PD in the absence or presence of 10 μM glycyrrhizin, isoliquiritigenin, or glycyrrhizin plus isoliquiritigenin (**i**) for 1 h prior to transduction with pSARS-CoV-2 in the presence of each drug. **j** Experimental timeline for YGS pretreatment before transduction with pSARS-CoV-2. **k**, **l** ACE2^+^ (**k**) and ACE2/TMPRSS2^+^ (**l**) cells were pretreated with serial threefold dilutions of YGS at a starting concentration of 100 mg/ml for 20 min four times at 4 h intervals and then transduced with pSARS-CoV-2 without YGS. **m** PG syrup, mainly made of PG root and frequently used for respiratory disease. **n**, **o** ACE2^+^ (**n**) and ACE2/TMPRSS2^+^ cells (**o**) were pretreated with serial threefold dilutions of a PG syrup stock solution containing 15 mg/ml of the PG root for 1 h prior to transduction with pSARS-CoV-2 in the presence of syrup. After culture for 24 h, the viral entry efficiency was quantified by measuring the activity of firefly luciferase in the cell lysates. The data were representative of two or three independent experiments with triplicate samples. The error bars indicate the SEM. *P* values were determined by the unpaired, two-tailed Student’s *t*-test. NS not significant.

To determine which of the two entry pathways is the target of PD, we prepared additional cell lines, H1299 and HEK293T, overexpressing both ACE2 and TMPRSS2 (ACE2/TMPRSS2^+^) and compared the inhibitory effects of various drugs with those on ACE2^+^ cells. We found that E64d and chloroquine, inhibitors of lysosomal cathepsins, effectively blocked pSARS-CoV-2 entry only in ACE2^+^ cells but not in ACE2/TMPRSS2^+^ cells, whereas camostat and nafamostat, inhibitors of TMPRSS2, effectively blocked pSARS-CoV-2 entry into ACE2/TMPRSS2^+^ cells but not into ACE2^+^ cells (Fig. 2a, b). In contrast, 5 μM PD completely inhibited pSARS-CoV-2 entry in both ACE2/TMPRSS2^+^ and ACE2^+^ cells (Fig. 2a, b). The actual potency of the PD inhibition of pSARS-CoV-2 entry into ACE2/TMPRSS2^+^ cells (as an IC_50_ value) was determined to be 0.72 μM (Fig. 2c), which was almost identical to that in ACE2^+^ cells (Fig. 1d). These results suggest that PD targets any one of the events that is common to both entry pathways.

Yonggaksan (YGS, Fig. 2d), in which the main ingredient is an extract powder from the PG root, has been available as an over-the-counter medicine and used for the treatment of phlegm, cough, and sore throat for more than 50 years in Korea and 200 years in Japan. Thus, we examined whether YGS has potential as a nonprescription medicine to block SARS-CoV-2 entry. We found that a dilute solution of YGS effectively inhibited pSARS-CoV-2 entry into both ACE2^+^ and ACE2/TMPRSS2^+^ cells in a dose-dependent manner, with similar IC_50_ values of 3.17 mg/ml in ACE2^+^ cells (Fig. 2e) and 3.72 mg/ml in ACE2/TMPRSS2^+^ cells (Fig. 2f), suggesting its therapeutic potential for SARS-CoV-2. Another main ingredient of YGS is an extract powder from licorice roots (active component is glycyrrhizin), which has the ability to inhibit the replication and penetration of SARS-CoV^27^ and is therefore thought to have therapeutic potential for COVID-19^28^. Thus, we tested the effects of glycyrrhizin and isoliquiritigenin, another major component of licorice roots, on the entry of pSARS-CoV-2. We found that both compounds exhibited no significant inhibitory activity against pSAR-CoV-2 entry into either ACE2^+^ or ACE2/TMPRSS2^+^ cells (Fig. 2g, h). Moreover, mixing the two compounds with 1 μM PD did not either enhance or reduce pSARS-CoV-2 entry into ACE2^+^ cells (Fig. 2i). Quantification of YGS components by high-performance liquid chromatography (HPLC) further revealed that PD is the main active compound of YGS that blocks pSARS-CoV-2 entry (Supplementary Information, Figs. S2, S3). The common daily dosage and regimen for YGS involves the application of one spoonful (0.3 g) of YGS powder to the throat surface without water, waiting for 20–30 min, and repeating 3–6 times a day. To mimic this usage, we treated the cells with YGS four times for 20 min each time at 4 h intervals before adding pSARS-CoV-2 (Fig. 2j). Under this dosage and regimen, YGS effectively reduced pSARS-CoV-2 entry, with similar IC_50_ values of 13.49 mg/ml in ACE2^+^ cells (Fig. 2k) and 15.26 mg/ml in ACE2/TMPRSS2^+^ cells (Fig. 2l). Taken together, these results suggest that YGS, through the action of PD, has high potential for the treatment of SARS-CoV-2 infection as a nonprescription, over-the-counter medicine.

In Korean culture, the root of PG is often served as a side dish at daily meals. For example, PG syrup (PGS) has been considered a folk remedy for relieving several symptoms of respiratory diseases. PGS (Fig. 2m) is prepared by chopping PG roots (Fig. 1a) into small pieces and marinating them in sugar or honey for several months and then mixing them with warm water and served as a drink. To test whether PGS also has an inhibitory effect on pSARS-CoV-2 entry, we treated ACE2^+^ and ACE2/TMPRSS2^+^ cells with serial threefold dilutions of PGS stock solution containing 15 mg/ml PG root for 1 h before infection with pSARS-CoV-2. We found that PGS effectively inhibited pSARS-CoV-2 entry, with similar IC_50_ values of 2.91 mg/ml (Fig. 2n) in ACE2^+^ cells and 3.69 mg/ml in ACE2/TMPRSS2^+^ cells (Fig. 2o). Moreover, HPLC analysis demonstrated that PD is the main active compound in PG roots that has inhibitory activity against pSARS-CoV-2 entry (Supplementary Information, Figs. S2, S3). These results suggest that even dietary foods containing PG roots might also have beneficial effects on COVID-19 patients.

### PD blocks SARS-CoV-2 entry by preventing cholesterol-dependent membrane fusion

**Fig. 3:**
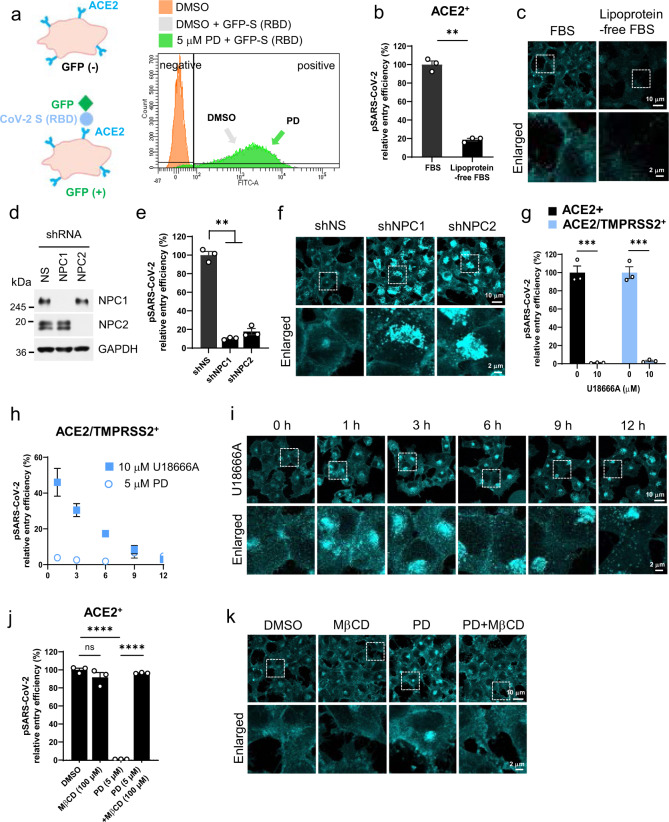
PD blocks SARS-CoV-2 entry by preventing cholesterol-dependent membrane fusion. **a** Schematic illustration showing that ACE2^+^ H1299 cells become GFP-positive when the GFP-conjugated SARS-CoV-2 spike receptor binding domain (RBD) (GFP-S (RBD)) are bound to ACE2 on host cells. **b**, **c** ACE2^+^ cells were incubated in culture medium containing 10% FBS or 10% lipoprotein-free FBS for 24 h prior to the pSARS-CoV-2 entry assay (**b**) or staining with filipin-III (**c**). **d–f** ACE2^+^ cells were infected with lentiviral particles expressing a nonspecific (NS) shRNA or an shRNA targeting *NPC1* or *NPC2* for 4 days. The protein levels of NPC1 and NPC2 were assessed by western blot. GAPDH was used as a loading control (**d**). The cells were used for the pSARS-CoV-2 entry assay (**e**) or filipin-III staining (**f**). **g** ACE2^+^ and ACE2/TMPRSS2^+^ cells were pretreated with 10 μM U18666A for 12 h prior to the pSARS-CoV-2 entry assay. **h**, **i** ACE2/TMPRSS2^+^ cells were pretreated with 10 μM U18666A or 5 μM PD for the indicated amount of time, followed by the pSARS-CoV-2 entry assay (**h**) or staining with filipin-III (**i**). **j**, **k** ACE2^+^ cells were treated with 5 μM PD in the presence or absence of 100 μM MβCD for 1 h, followed by the pSARS-CoV-2 entry assay (**j**) or staining with filipin-III (**k**). The data from the pSARS-CoV2 entry assay are representative of two or three independent experiments with triplicate samples. The error bars indicate the SEM. *P* values were determined by the unpaired, two-tailed Student’s *t*-test (**b**, **e**, **g**) or one-way analysis of variance (ANOVA) followed by Tukey’s post hoc test (**j**). *P < 0.05; **P < 0.01; ***P < 0.001; ****P < 0.0001; NS not significant. The error bars are not visible when they are within the symbols (**h**).

To delineate the detailed molecular and cellular mechanisms of PD action, we explored two possible events that are common to both entry pathways: (1) the initial binding of the S protein to ACE2 at the plasma membrane and (2) the fusion of the viral membrane to the host cell membrane for the translocation of viral RNA into the cytosol of host cells. Of the two events, we tested the first possibility. The S protein of SARS-CoV-2 binds to ACE2 via a receptor binding domain (RBD) in the S1 subunit^29^. ACE2^+^ cells were incubated with medium containing the RBD of SARS-CoV-2 fused to GFP (S (RBD)-GFP). Flow cytometry analysis showed that S (RBD)-GFP was capable of binding over 95% of ACE2^+^ cells. Importantly, pretreatment with 5 μM PD did not change the ability of S (RBD)-GFP to bind to ACE2^+^ cells (Fig. 3a). Thus, we eliminated the possibility that PD influences a specific interaction between the SARS-CoV-2 S protein and ACE2. We then investigated the possible mechanism of PD action during membrane fusion. It has been reported that PD helps lower cholesterol levels in a mouse model of hypercholesterolemia^30^ and that depletion of membrane cholesterol content inhibits TMPRSS2-mediated coronavirus fusion^31–33^, raising the possibility that PD directly influences the membrane cholesterol content to disturb membrane fusion. To elucidate the role of cholesterol in SARS-CoV-2 entry, we performed an entry assay with ACE2^+^ cells grown in lipoprotein-free culture medium and compared the results to those obtained with control medium. Lipoprotein-free culture conditions caused a significant reduction in pSARS-CoV-2 entry into ACE2^+^ cells (Fig. 3b) and a significant depletion of cholesterol on the plasma membrane as well as in other intracellular compartments, as revealed by the staining of ACE2^+^ cells with filipin-III, a fluorescent dye that binds to free cholesterol (Fig. 3c). These results support that the inhibitory action of PD on SARS-CoV-2 entry could be due to its effect on membrane cholesterol.

As another example of disrupted cholesterol trafficking, we explored the cellular model of Niemann–Pick type C (NPC), which is characterized by a redistribution of free cholesterol from the plasma membrane and accumulation in the late endosome and lysosome compartment^34^. NPC is caused by loss-of-function mutations in either the *NPC1* or *NPC2* gene^35^. After generating *NPC1-* and *NPC2*-specific shRNAs (Fig. 3d), we performed a pSARS-CoV-2 entry assay and found that the gene silencing of *NPC1* and *NPC2* by the respective shRNAs significantly reduced the pSARS-CoV-2 entry into ACE^+^ cells (Fig. 3e). Staining with filipin-III showed the expected altered trafficking of membrane cholesterol and the accumulation of endolysosomal cholesterol (Fig. 3f). We next utilized U18666A, a well-known inhibitor of NPC1, to pharmacologically disturb membrane cholesterol. U18666A is known to inhibit NPC1 function by directly binding to the sterol-sensing domain of the NPC1 protein^36^. Furthermore, treatment with U18666A has been shown to disrupt the entry of a variety of enveloped viruses, including dengue virus, Ebola virus, HCV, influenza A virus, Zika virus, chikungunya virus, and HIV^37^, as well as coronaviruses such as SARS-CoV, Middle East respiratory syndrome-related coronavirus (MERS-CoV), and Type I feline coronavirus (F-CoV)^38,39^. We found that 12 h of treatment with 10 μM U18666A completely blocked pSARS-CoV-2 entry into both ACE2^+^ and ACE2/TMPRSS2^+^ cells (Fig. 3g). Intriguingly, the slow time course of the blocking effect of U18666A on pSARS-CoV-2 entry into ACE2/TMPRSS2^+^ cells (Fig. 3h) coincided very well with the slow time course of the disappearance of membrane cholesterol (Fig. 3i). This slow time course of U18666A was in marked contrast to the fast-acting effect of PD (Fig. 3h). These results suggest that disturbing membrane cholesterol can be an effective target to block SARS-CoV-2 entry.

To test whether PD blocks SARS-CoV-2 entry by disrupting membrane cholesterol, we utilized methyl β-cyclodextrin (MβCD), which exhibits a doughnut-shaped structure that makes it a natural complexing agent for encapsulating cholesterol and other large molecules, such as hormones, vitamins, and natural hydrophobic macrocompounds^40^. When cells are exposed to high concentrations of MβCD (5–10 mM) for more than 2 h, 80–90% of total cellular cholesterol can be removed^41^. However, at much lower concentrations and a 1-h incubation time, MβCD should not encapsulate cholesterol. Indeed, after incubation at 100 μM for 1 h, MβCD treatment showed no significant effect on pSARS-CoV-2 entry into ACE2^+^ cells (Fig. 3j). Under the same conditions, MβCD completely reversed the blocking effect of PD on pSARS-CoV-2 entry to the level of the control DMSO (Fig. 3j). PD alone induced a dramatic redistribution of membrane and endolysosomal cholesterol (Fig. 3k), which was somewhat different from the effect of U18666A (Fig. 3i, 12 h). These results indicate that 100 μM MβCD completely sequestered 5 μM PD without affecting the membrane cholesterol content. Indeed, staining with filipin-III showed no alteration of membrane cholesterol after treatment with 100 μM MβCD alone, a significant redistribution of membrane cholesterol after treatment with 5 μM PD, and complete reversion of membrane cholesterol after treatment with 100 μM MβCD together with 5 μM PD (Fig. 3k).

**Fig. 4:**
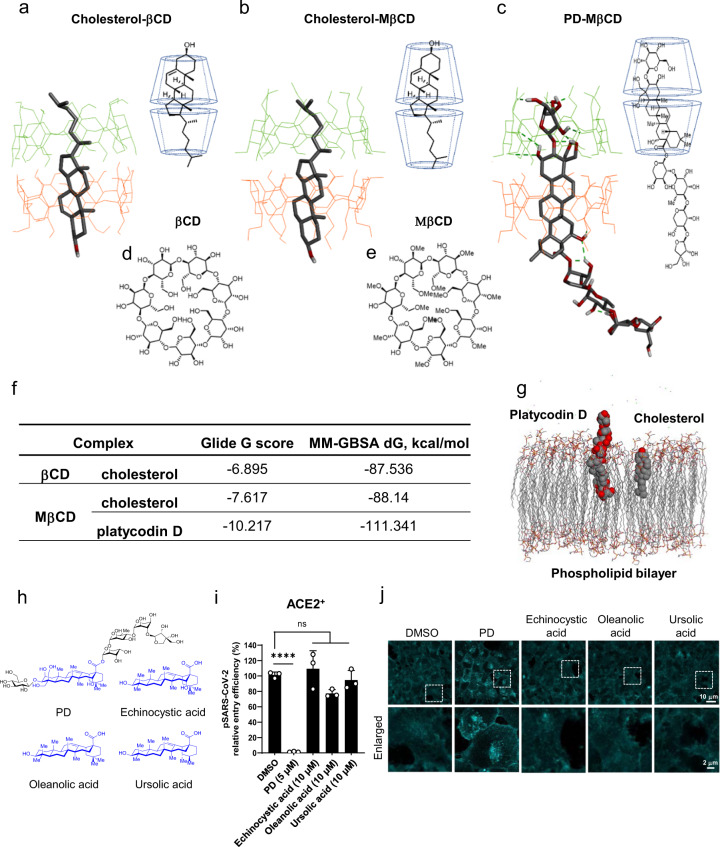
The encapsulation of PD by MβCD and the significantly higher affinity of MβCD to PD than to cholesterol. **a** Energy-minimized cholesterol-βCD crystal structure. **b**, **c** Cholesterol-MβCD inclusion complex, modeled based on the βCD template (**b**). PD-MβCD inclusion complex (**c**). The guest ligand is shown in stick form, whereas the host molecules are presented as green and orange lines. Hydrogen bonds (both intramolecular and those between the host and guest molecules) are depicted as green dashed lines. Only polar hydrogens are shown for image clarity. **d, e** Chemical structures of βCD (**d**) and tetradeca-2,6-O-methyl-β-cyclodextrin (**e**). **f** Glide scores and binding affinity calculations for the βCD inclusion complexes. **g** Model of PD and cholesterol in a POPE explicit membrane. **h** Comparison of the chemical structure of platycodin D with those of natural triterpene compounds without sugar moieties used in this experiment. **i** ACE2^+^ cells were pretreated with 5 μM PD or 10 μM echinocystic acid, oleanolic acid, and ursolic acid for 1 h prior to the pSARS-CoV-2 entry assay. The data were representative of three independent experiments with triplicate samples. The error bars indicate the SEM. *P* values were determined by the unpaired, two-tailed Student’s *t*-test. NS not significant. **j** ACE2^+^ cells were treated with 5 μM PD or 10 μM of each compound as indicated for 24 h and then stained with filipin-III.

To understand the detailed molecular mechanisms by which PD prevents pSARS-CoV-2 entry at the chemical structure level, we utilized dynamic molecular modeling techniques. Based on the previously reported crystal structure of the cholesterol-βCD encapsulation complex (Fig. 4a)^42^, we performed homology modeling of cholesterol-MβCD and PD-MβCD complexes (Fig. 4b, c). We found that out of 21 hydroxyl groups in βCD (Fig. 4d), ~12–18 could be methylated at different positions, while MβCD with 14 methyl groups showed the greatest encapsulating capacity. Therefore, we chose tetradeca-2,6-*O*-methyl-β-cyclodextrin (Fig. 4e) as the representative MβCD to model the cholesterol-MβCD and PD-MβCD complexes and calculated the binding energy for each molecular pair in terms of the glide G-score and the molecular mechanics/generalized born surface area (MM-GBSA) free energy scores (Fig. 4f). The cholesterol-MβCD encapsulation complex was formed by two MβCD molecules arranged coaxially, forming a head-to-head dimer via intramolecular hydrogen bonds and one cholesterol molecule fitted snugly into the hydrophobic cavity of MβCD, whose methoxymethyl groups encapsulated cholesterol better than the cholesterol-βCD complex (Fig. 4b). In the PD-MβCD encapsulation complex, the hydrophobic core of PD fit well into the MβCD host dimer, whereas the hydrophilic sugar tail of PD was fully exposed to the solvent (Fig. 4c). Overall, the molecular modeling of MβCD, PD, and cholesterol revealed that a homodimer of MβCD snugly encapsulated PD and cholesterol with a higher affinity to PD than cholesterol (Fig. 4f), supporting the possibility that MβCD preferentially sequesters PD over cholesterol. A similar molecular modeling of membrane-inserted PD and cholesterol in an explicit lipid bilayer model also predicted the similar orientations of the partly embedded PD and fully embedded cholesterol (Fig. 4g). In particular, unlike cholesterol, the hydrophilic sugar moiety of PD stuck out of the lipid bilayer (Fig. 4g), creating a protrusion on the surface of the membrane, which might have the ability to hinder membrane fusion. Furthermore, none of the other natural triterpene compounds without a sugar moiety but with a backbone structure similar to that of PD, including echinocystic acid, oleanolic acid, and ursolic acid (Fig. 4h), showed inhibitory activity against pSARS-CoV-2 entry (Fig. 4i) and altered the distribution of membrane cholesterol (Fig. 4j), implying that the sugar moieties attached to the saponin backbone of PD might be responsible for its action against pSARS-CoV-2 entry by altering membrane cholesterol. These results raise the strong possibility that PD prevents membrane fusion in a cholesterol-dependent manner.

### PD inhibits exocytosis-mediated membrane fusion

**Fig. 5:**
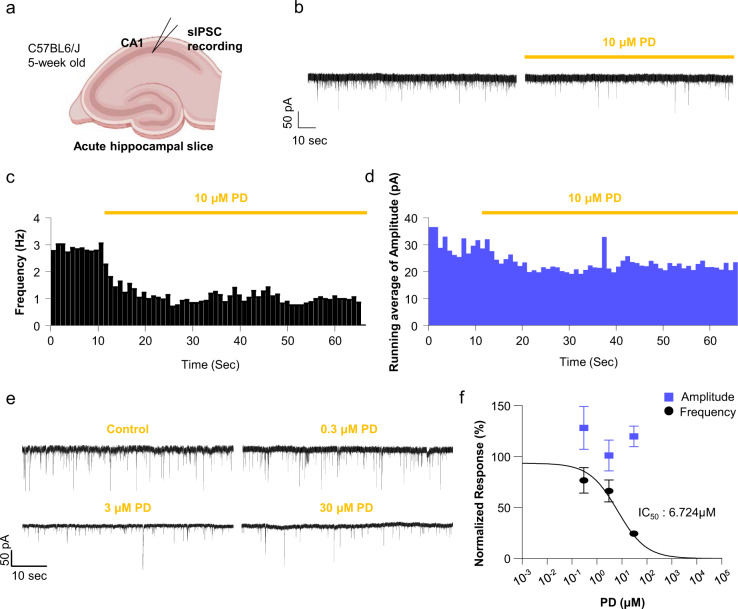
PD prevents membrane fusion, as evidenced by the reduced frequency of spontaneous inhibitory postsynaptic currents (sIPSCs) in CA1 neurons. **a** Experimental schematic of sIPSC recording in the CA1 hippocampus. **b** Representative traces of sIPSCs recorded from the CA1 hippocampus before 10 μM PD treatment (left) and during 10 μM PD bath application (right). **c** Running frequency during 1 h of 10 μM PD treatment. sIPSC events were binned for 1 min to represent the frequency in Hz. **d** Running average amplitude during 1 h of 10 μM PD treatment. sIPSC amplitudes were binned for every minute. **e** Representative traces of sIPSCs recorded from the CA1 hippocampus in control, 0.3, 3, and 30 μM PD-treated slices. **f** Normalized responses for the frequencies (black) and amplitudes (blue) of sIPSCs in the control (*n* = 15), 0.3 μM (*n* = 8), 3 μM (*n* = 9), and 30 μM (*n* = 6) PD treatment groups. The control condition data (0 μM) were not represented but were included in the nonlinear fitting.

We next tested the effect of PD on the well-known membrane fusion event of spontaneous inhibitory postsynaptic currents (sIPSCs) in acute brain slices as a proof-of-concept experiment and a proxy methodology for monitoring fusion of the viral envelope with the host cell membrane. We performed slice patch-clamp recordings of hippocampal CA1 pyramidal neurons (Fig. 5a) and recorded sIPSCs before and during the application of PD (Fig. 5b). We found that PD effectively inhibited the frequency of sIPSCs without affecting the amplitude (Fig. 5c, d). The time course of PD inhibition was relatively fast, occurring ~10–15 s after onset (Fig. 5c). The concentration-response relationship showed that PD effectively inhibited the sIPSC frequency, with an IC_50_ of 6.724 μM, whereas PD did not inhibit the amplitude of sIPSCs (Fig. 5e, f). These results support that the inhibitory action of PD on pSARS-CoV-2 entry might be due to its ability to interfere with membrane fusion. While whether an intermolecular relationship exists between PD and cholesterol remains unknown, cholesterol appears to help facilitate the incorporation of PD into the membrane, as evidenced by a 2.5-fold increase in the IC_50_ value for the inhibition of pSARS-CoV-2 entry under lipoprotein-free conditions (Supplementary Information, Fig. S4). Taken together, these results strongly suggest that PD blocks SARS-CoV-2 entry by interacting with cholesterol and preventing fusion of the viral membrane to the host cell membrane.

### PD inhibits the authentic SARS-CoV-2 infection of Vero and Calu-3 cells

**Fig. 6:**
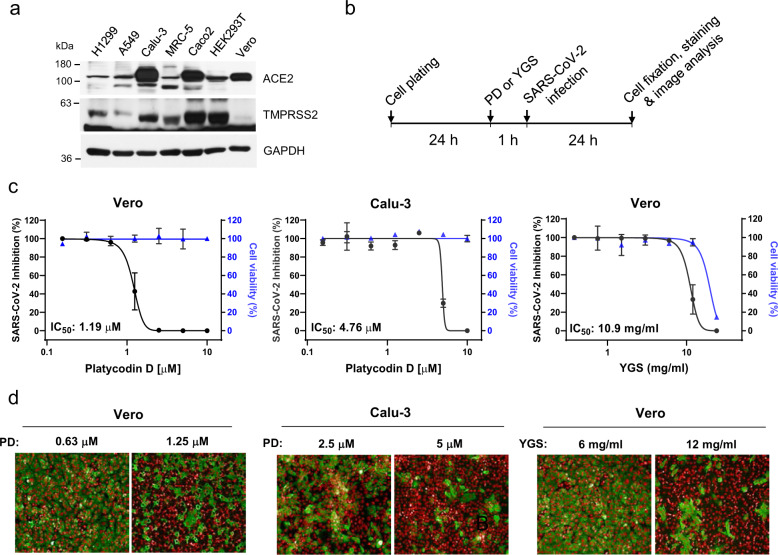
PD inhibits the authentic SARS-CoV-2 infection of Vero and Calu-3 cells. **a** The protein levels of ACE2 and TMPRSS2 in multiple cell lines were assessed by western blot. GAPDH was used as a loading control. **b** Experimental timeline for PD or YGS treatment and authentic SARS-CoV-2 infection before staining for the viral N protein and image analysis. **c** Dose-response curve immunofluorescence analysis of PD and YGS. The black circles represent inhibition of authentic SARS-CoV-2 infection (%), and the blue triangles represent cell viability (%). The mean ± SEM was calculated from duplicate experiments. **d** Confocal microscopy images showing the viral N protein (green) and cell nuclei (red) at concentrations near the IC_50_ of PD in Vero and Calu-3 cells and near the IC_50_ of YGS in Vero cells.

**Fig. 7:**
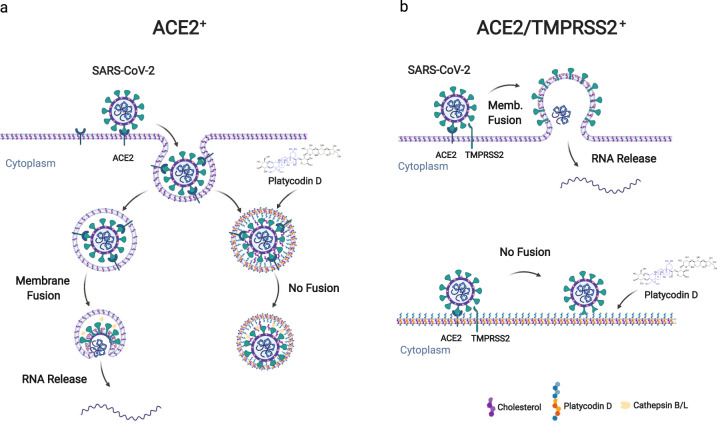
Schematic model of the mechanism by which PD prevents SARS-CoV-2 entry. **a**, **b** In both ACE2^+^ and ACE2/TMPRSS2^+^ cells, PD incorporates into the host membrane with sugar moieties protruding through the surface of the membrane and inhibits SARS-CoV-2 membrane fusion with the endolysosomal membrane (**a**) and the plasma membrane (**b**).

As a final measure, we evaluated the antiviral activity of PD on authentic SARS-CoV-2 viruses obtained from the Korea Centers for Disease Control and Prevention (KCDC). We performed an immunocytochemistry-based assessment of SARS-CoV-2 infection using an antibody against the SARS-CoV-2 nucleocapsid N protein, as previously described^9^. Infected host cells were automatically counted using in-house image analysis software. For the host cells, we utilized both the monkey-derived Vero and the human-derived Calu-3 cell lines. Western blot analysis showed abundant expression of ACE2 in both cell types, the lack of TMPRSS2 expression in Vero cells, and high expression of TMPRSS2 in Calu-3 cells (Fig. 6a). We found that PD significantly reduced SARS-CoV-2 infection in both TMPRSS2-negative Vero cells and TMPRSS2-positive Calu-3 cells, with IC_50_ values of 1.19 and 4.76 μM, respectively (Fig. 6b–d). YGS also effectively inhibited the SARS-CoV-2 infection of Vero cells, with an IC_50_ of 10.9 mg/ml (Fig. 6c, d). These IC_50_ values obtained from authentic SARS-CoV-2 viruses were within a range similar to those obtained from pSARS-CoV-2 (Figs. 1c, 2c), indicating that PD is equally effective against authentic SARS-CoV-2 viruses. Unlike other previously reported drugs (Table 1), PD shows uniquely exhibits equally high potencies against SARS-CoV-2 infection of both TMPRSS-negative Vero and TMPRSS-positive Calu-3 cells. This is in marked contrast to other drugs, which show only one-sided potency in either TMPRSS-negative Vero (chloroquine) or TMPRSS-positive Calu-3 (camostat, nafamostat). Taken together, these results indicate that PD has an important advantage over other known drugs in that it can potently prevent SARS-CoV-2 infection by inhibiting both lysosome- and TMPRSS2-driven SARS-CoV-2 entry pathways during the common process of viral membrane fusion by disrupting membrane cholesterol on the host cell (Fig. 7).

**Table 1:** List of drugs with IC_50_ in Vero and Calu-3 cells.

Drug	IC_50_ in Vero (μM)	IC_50_ in Calu3 (μM)
**platycodin D**	1.19	4.76
**YGS**	10.9 mg/ml	n.d.
**chloroquine**	7.91	>50^a^
**camostat**	>50^a^	0.187^a^
**nafamostat**	13.88^a^	0.0022^a^
**remdesvir**	10.67	1.74

^a^Ref. ^9^.

## Discussion

SARS-CoV-2 binds to ACE2 and enters host cells via membrane fusion after proteolytic cleavage of the SARS-CoV-2 S protein by (1) cathepsin B/L in the lysosomal membrane or (2) TMPRSS2 in the plasma membrane. We have demonstrated that PD effectively inhibits both of the SARS-CoV-2 entry pathways almost equally in ACE^+^ and ACE/TMPRSS2^+^ cells, with IC_50_ values ranging from 0.69 to 4.76 μM against both pseudo and authentic SARS-CoV-2 viruses. To the best of our knowledge, PD is the first single compound that simultaneously blocks the two main pathways of SARS-CoV-2 infection. As a first-generation inhibitor, chloroquine was introduced as a potential inhibitor against SARS-CoV-2 infection. However, clinical trials with this drug have mostly failed because it blocks only cathepsin-mediated viral entry^7^. To date, TMPRSS2 inhibitors such as camostat and nafamostat have been considered as second-generation inhibitors of SARS-CoV-2 entry. However, they were recently reported to be ineffective at preventing viral entry into TMPRSS2-negative cells^8,9^. Thus, we propose PD as the next-generation inhibitor of SARS-CoV-2 infection, as it blocks both entry pathways with much higher potency than existing drug candidates, such as chloroquine, camostat, and nafamostat.

One of the highlights of our study is that PD alters the distribution of membrane cholesterol, which contributes to its anti-SARS-CoV-2 activity. Our finding is consistent with very recent reports showing that 25-hydrocholesterol (25-HC) has an antiviral effect on SARS-CoV-2 infection by promoting the accumulation of cholesterol in late endosomes and potentially restricting S protein-mediated membrane fusion via the depletion of membrane cholesterol^31–33^. These reports strongly support our notion that alteration of membrane cholesterol can be an effective strategy to prevent SARS-CoV-2 infection. However, how PD interacts with membrane cholesterol and inhibits membrane fusion remains unclear. Although pharmacological inhibition of NPC caused a depletion of membrane cholesterol (Fig. 3i), PD did not deplete membrane cholesterol (Fig. 3k). These results raise the possibility that the PD mechanism is independent of cholesterol. On the other hand, the PD potency decreased by 2.5-fold when cholesterol was depleted (Supplementary Information, Fig. S4), suggesting that cholesterol helps to facilitate the intercalation of PD into the membrane. Molecular modeling experiments suggest that PD and cholesterol behave similarly because the hydrophobic triterpenoid saponin moieties of PD and cholesterol are similar in size and hydrophobicity. The major structural difference derives from the fact that PD contains an additional elaborate sugar moiety that is strongly hydrophilic due to the multiple hydroxyl groups of the sugar moiety, which cholesterol lacks. This raises an interesting possibility that while PD behaves similarly to cholesterol within the lipid bilayer, PD behaves profoundly differently outside the lipid bilayer, creating a physical hindrance due to the elaborate sugar tail that extends out from the membrane (Fig. 4g). Considering that the thickness of the lipid bilayer is ~10 nm, the sugar tail of PD could extend out by ~2–3 nm (Fig. 4g). Such conspicuous protrusions on the membrane could greatly hinder any membrane fusion events that require close proximity and direct contact between two membrane structures, e.g., the SARS-CoV-2 membrane and the host cell membrane. A classic example of such fusion is synaptic release due to fusion of the synaptic vesicle membrane to the presynaptic terminal membrane during exocytosis. Indeed, we found that PD could also inhibit synaptic release events due to membrane fusion (Fig. 5), supporting our hypothesis that the PD sugar tail protrusion hinders membrane fusion. Future investigations are needed to systematically test this exciting possibility.

Recent studies have revealed that ACE2 and TMPRSS2 are highly expressed in nasal epithelial cells and at lower levels in lung tissues^43,44^. These reports provide a plausible explanation for why viral loads of SARS-CoV-2 in the upper respiratory tract peak before the onset of symptoms and why presymptomatic transmission occurs. Therefore, reducing the viral loads in the upper respiratory tracts of COVID-19 patients at the early and asymptomatic stages must be the most effective strategy to stop the current pandemic. Here, we tested the anti-SARS-CoV-2 infection activity of YGS, a commercially available herbal medicine containing PG as the main ingredient, and obtained IC_50_ values of 3.17 and 3.72 mg/ml for ACE2^+^ and ACE2/TMPRSS2^+^ cells infected with pSARS-CoV-2, respectively, and a value of 10.9 mg/ml for Vero cells infected with authentic SARS-CoV-2. YGS is formulated as a fine powder, and traditional usage involves the application of a spoonful (300 mg) of YGS onto the throat membrane and waiting for several minutes without drinking water. Through this unique formulation and usage, an effective PD concentration can be reached at regions around the throat mucus membrane and along the epithelial lining of the upper respiratory airways. Alternatively, if applicable, PD can be easily and directly administered via the nasal route in the form of drops or spray for therapeutic purposes. These ideas should be tested immediately in animal models and COVID-19 patients. Therefore, we suggest that PG-containing herbal medicines and foods as well as the PD compound itself are promising therapeutic options for halting the spread of SARS-CoV-2 within and between individuals.

It has been predicted that the emergence of SARS-CoV-2 from bat-originated SARS-CoV is probably due to naturally occurring mutations during its natural course of spread^45^. Such an alarming concept forebodes further emergence of more virulent versions of SARS-CoV-2. However, our study supports that Mother Nature has already prepared natural products, such as PD, that are capable of protecting humans from SARS-CoV-2 infection, which is comforting. This raises the important point that it might be advantageous to find a cure among vastly diverse natural products in addition to developing vaccines, which might become ineffective upon the emergence of new SARS-CoV-2 variants^45^.

## Materials and Methods

### Cell culture

H1299, A549, MRC-5, and Caco2 cells were obtained from the Korean Cell Line Bank. HEK293T cells were obtained from the American Type Culture Collection (ATCC, USA, CRL-3216). H1299 and A549 cells were cultured in RPMI-1640 (Gibco, USA), and MRC-5, Caco2, and HEK293T cells were cultured in Dulbecco’s modified Eagle’s medium (DMEM; Corning, USA) supplemented with 10% fetal bovine serum (FBS) (Gibco, USA) and 1X penicillin-streptomycin solution (HyClone, USA) at 37 °C in a humidified incubator containing 5% CO2. For cell culture under lipoprotein-free conditions, lipoprotein-depleted 10% FBS (Kalen Biomedical, USA) was used. For authentic SARS-CoV-2 experiments, Vero cells were obtained from ATCC (CCL-81) and maintained at 37 °C with 5% CO2 in DMEM supplemented with 10% FBS and 1X antibiotic-antimycotic solution (Gibco, USA). The Calu-3 cells used in this study were derived from a clonal isolate that grew faster than the parental Calu-3 cells obtained from ATCC (HTB-55). Calu-3 cells were maintained at 37 °C with 5% CO2 in Eagle’s Minimum Essential Medium (EMEM, ATCC) supplemented with 10% FBS and 1X antibiotic-antimycotic solution.

### Reagents

PD, U18666A, E64d, chloroquine, camostat mesylate, nafamostat mesylate, ginsenoside Rb1, ginsenoside Rg3, ginsenoside mix, glycyrrhizin, isoliquiritigenin, echinocystic acid, oleanolic acid, ursolic acid, and methyl-beta-cyclodextrin were purchased from Sigma-Aldrich Co. (USA).

### Plasmids and establishment of stable cell lines

The full-length ACE2 sequence from pCEP4-myc-ACE2 (a gift from Erik Procko, Addgene plasmid #141185) was cloned into the pHR-CMV lentiviral expression vector (a gift from A. Radu Aricescu, Addgene plasmid # 113888) via the EcoRI and AgeI restriction sites. The TMPRSS2 lentiviral expression vector, RRL.sin.cPPT. SFFV/TMPRSS2 (variant 1).IRES-neo. WPRE (MT130), was gifted by Caroline Goujon (Addgene plasmid # 145843). For gene silencing, oligonucleotides that contained shRNA sequences targeting NPC1 (5′-CCAGGTTCTTGACTTACAA-3′) and NPC2 (5′-CGGTTCTGTGGATGGAGTTAT-3′) and nonspecific a (NS) sequence (5′-CAACAAGATGAAGAGCACCAA-3′) were cloned into the pLKO.1 puro lentiviral shRNA plasmid. For stable cell line generation, these plasmids were transiently transfected into HEK293T cells with the packaging plasmid psPAX2 and envelope plasmid pMD2.G using Lipofectamine 3000 transfection reagent (Thermo Fisher, USA) according to the manufacturer’s instructions. At 24 and 48 h posttransfection, the lentivirus particles containing supernatants were harvested, filtered through 0.45-μm-pore size filters and used to infect H1299 cells, which were seeded on 6-well plates and cultured until reaching 70–80% confluence. One milliliter of the virus supernatant was directly overlaid onto the cells in the presence of polybrene (Merck, Germany) at a final concentration of 4 μg/ml. After 24 h, the supernatant medium was replaced with fresh medium, and the cells were cultured for 2–3 days.

### SARS-CoV-2 spike-pseudotyped lentivirus production

SARS-CoV-2 spike (S)-pseudotyped lentiviruses were generated using a second generation lentiviral packing system. In brief, HEK293T cells at 70–80% confluency in a 6-well plate were transfected with 1 μg of a lentiviral backbone that contained expression cassettes for firefly luciferase, 0.75 μg of the psPAX2 packing plasmid, and 0.5 μg of the SARS-CoV-2 spike plasmid (a gift from Fang Li, Addgene plasmid #145032) using the Lipofectamine 3000 transfection reagent (Invitrogen, USA) according to the manufacturer’s instructions. The pMD2.G plasmid was used to create a control lentivirus pseudotyped with VSV-G. At 24 and 48 h posttransfection, supernatants containing SARS-CoV-2 S-pseudotyped virus particles were collected, filtered through a 0.45-µm-pore size filter, and stored at 4 °C until use.

### pSARS-CoV-2 entry assay

H1299 cells stably expressing ACE2 or ACE2 together with TMPRSS2 were cultured in 48-well plates until reaching 70–80% confluence. To determine the effect of drugs on pSARS-CoV-2 entry, stable H1299 cells were pretreated for 1 h with each drug and then overlaid with virus-containing supernatants in the presence of each drug. After 24 h incubation, the viral entry efficiency was quantified by measuring the activity of firefly luciferase in cell lysates using a luciferase assay system (Promega, USA) and the SpectraMax iD5 Multi-Mode Microplate Reader (Molecular Devices, USA).

### Authentic SARS-CoV-2 virus and dose-response curve analysis by the immunofluorescence assay

The Korean strain of SARS-CoV-2 (βCoV/KOR/KCDC03/2020) was provided by the KCDC and propagated in Vero cells. Viral titers were determined by plaque assays in Vero cells. All experiments using SARS-CoV-2 were performed at the Institut Pasteur Korea in compliance with the guidelines of the KNIH using enhanced biosafety level 3 (BSL-3) containment procedures in laboratories approved for use by the KCDC. Vero cells were seeded at 1.2 × 10^4^ cells per well, and Calu-3 cells were seeded at 2.0 × 10^4^ cells per well in black, 384-well, μClear plates (Greiner Bio-One, Austria) 24 h prior to the experiment. Ten point DRCs were generated, with compound concentrations ranging from 0.02–10 μM and 0.05–24 mg/ml except for chloroquine and remdesivir, which ranged from 0.1–150 μM. For viral infection, plates were transferred into the BSL-3 containment facility, and SARS-CoV-2 was added at a multiplicity of infection (MOI) of 0.0125 in Vero cells and 0.5 in Calu-3 cells. The cells were fixed at 24 hpi with 4% paraformaldehyde (PFA), stained with the SARS-CoV-2 nucleocapsid (N) protein, and analyzed by immunofluorescence. The acquired images were analyzed using Columbus software (PerkinElmer, Inc. Waltham, MA) to quantify the cell numbers and infection ratios, and antiviral activity was normalized to positive (mock) and negative (0.5% DMSO) controls in each assay plate. DRCs were fitted by sigmoidal dose-response models using XLfit 4 or Prism6 software with the following equation: Y = Bottom + (Top ˗ Bottom)/(1 + (IC_50_/X)Hillslope). The half-maximal inhibitory concentration (IC_50_) values were calculated from the normalized activity dataset-fitted curves. IC_50_ values were measured in duplicate, and the quality of each assay was controlled by the Z′-factor and the coefficient of variation as a percentage (%CV).

### Cell viability assay

H1299, Calu-3, and Vero cells seeded in 96-well plates (5 × 10^3^ cells/well) were treated with the indicated concentrations of PD. After 24 h of treatment, WST-8 solution (Biomax, Korea) was added and incubated for 2 h. Water-soluble formazan formed in the culture medium was measured by a SpectraMax iD5 Multi-Mode Microplate Reader (Molecular Devices, USA) at an absorbance of 450 nm. The relative cell viability (%) is expressed as the percentage relative to the DMSO-treated control cells.

### Protein extraction and immunoblot analysis

Cell lysates were prepared by solubilizing cells with ice-cold RIPA lysis buffer (Rockland Immunochemicals, USA) supplemented with a protease and phosphatase inhibitor cocktail (Thermo Fisher Scientific, USA), followed by centrifugation at 13,000 rpm for 20 min. The cleared cell lysates were mixed with a proper volume of 5x SDS sample buffer, separated by SDS-PAGE, and transferred onto nitrocellulose membranes. After blocking with 5% skim milk in TBST (20 mM Tris-HCl (pH 7.5), 150 mM NaCl, 0.05% Tween 20) for 1 h at room temperature (RT), the membranes were incubated in TBST at 4 °C overnight with the following primary antibodies: rabbit anti-NPC1 (Novus, NB400-148), rabbit anti-NPC2 (Novus, NBP1-84012), rabbit anti-ACE2 (Abcam, ab15348), rabbit anti-TMPRSS2 (Abcam, ab92323), and mouse anti-GAPDH (Abcam, ab8245). The membranes were incubated with the corresponding horseradish peroxidase-conjugated secondary antibodies (KPL, USA) for 1 h at RT. Antibody-protein complexes were detected using ECL Western Blotting Substrate (Thermo Fisher Scientific, USA).

### Filipin III cholesterol staining

H1299 cells grown on coverslips were fixed with 4% PFA at RT for 10 min and then stained with 5 μg/ml filipin-III (Cayman, USA) in PBS/1% FBS solution at RT for 2 h in the dark. The stained cells were examined under an LSM700 confocal microscope (Carl Zeiss, Germany).

### Flow cytometry analysis

Expi293F cells were transfected with pcDNA3-SARS-CoV-2-S-RBD-sfGFP (a gift from Erik Procko, Addgene plasmid # 141184) using the ExpiFectamine™ 293 Transfection Kit according to the manufacturer’s directions (Thermo Fisher Scientific, USA). The cells were then cultured for 4–5 days and removed by centrifugation at 800×*g* for 5 min, and the culture medium was stored at −4 °C. To analyze the effects of PD on the binding of CoV-2-RBD-GFP to ACE2 on the cell surface, H1299 cells expressing ACE2 were treated with DMSO or 5 μM PD for 1 h and washed with ice-cold PBS-BSA, followed by incubation with a 1/10 dilution of medium containing CoV-2-RBD-GFP for 30 min on ice. H1299 cells were then washed twice with PBS-BSA and analyzed on a BD LSRFortessa™ flow cytometer.

### HPLC analysis

The PG root or YGS powder was dissolved in distilled water and analyzed using an HPLC system (Agilent 1100) at 203 nm with an RS Tech HECTOR-M C18 column (4.6 × 250 mm, 5 micron particle size, RS Tech Corp, Cheongju, South Korea). Elution was performed at 25 °C with a mixture of solvent A (water) and solvent B (acetonitrile). The gradient eluent system consisted of 82:18 (A:B) from 0 to 22 min, 82:18 (A:B) to 70:30 (A:B) from 22 min to 32 min, and 70:30 (A:B) to 50:50 (A:B) from 32 min to 60 min and a flow rate of 1 mL/min.

### Molecular modeling of MβCD inclusion complexes

The Schrodinger Maestro software 2017 suite (Maestro, Schrödinger, LLC, New York, NY) was used to prepare and score the MβCD binding complexes. The software tools were applied using the default settings at pH 7.4 unless stated otherwise below. The binding complex models were prepared based on the crystal structure of the β-cyclodextrin (βCD) cholesterol inclusion complex (CSD entry: KEXQUC)^42^. The crystal structure of tetradeca-2,6-*O*-methyl-β-cyclodextrin (CSD entry: BOYFOK03)^46^ was used to represent MβCD in the binding complex. Both crystal structures were obtained from the Cambridge Structural Database (CSD), and all water molecules were removed. The ligands cholesterol and PD were drawn in ChemDraw Professional 16.0 and imported as structure data files into the Maestro LigPrep module. The Ligprep module was used to prepare all ligands for further use and to determine the ligand partial charges, optimize the geometry, and perform energy minimization using the OPLS3 force field. The MβCD binding complexes were prepared using the βCD-cholesterol complex as a template. First, the atomic coordinates of the two template βCD units were copied onto two corresponding MβCD units. Cholesterol was added to the interior of the complex by copying the atomic coordinates of the template guest cholesterol molecule, whereas PD was placed using a rigid alignment, maximum common structure molecular overlay tool. To account for the increase in the macrocycle size, as well as to remove molecular strain and atomic bumps and relax the binding complex structures, a restrained energy minimization algorithm was applied using the OPLS3 force field and a heavy atom RMSD restriction of 2 Å. The obtained ligand binding poses were scored in place by the Glide SP (standard precision) dock score, and afterwards, the relative ligand binding affinity was estimated by the Prime MM-GBSA tool with a variable-dielectric generalized Born (VDGB) continuum solvation model and the OPLS3 force field. The binding affinity was calculated using the equation: ∆G_bind_ = E_complex(minimized)_ − (E_ligand(minimized)_ + E_host(minimized)_). More negative scores indicated stronger binding. Binding complex visualization was performed using the Discovery Studio Client 2020 package (Dassault Systèmes; BIOVIA. Discovery Studio Modeling Environment; Release 2020; Dassault Systèmes: USA, 2020).

### PD and cholesterol orientation in the cell membrane

The Discovery Studio Client 2020 package was used to optimize the position and orientation of PD and cholesterol in an explicit membrane bilayer consisting of 1-palmitoyl-2-oleoyl-sn-glycero-3-phosphoethanolamine (POPE) lipid molecules. Before running the membrane addition and molecule orientation algorithm, the 3D conformations of PD and cholesterol were prepared using the standard ligand preparation protocol and energy minimized with the general CHARMm force field. The molecule was first reoriented toward an implicit membrane by performing a stepwise search for the minimum solvation energy of the molecule, which was calculated by the CHARMM36 force field and the generalized Born implicit membrane (GBIM) module. The membrane thickness was set to 35 Å. After determining the optimal molecular position in the implicit membrane, the lipid bilayer was constructed by adding POPE lipid molecules, water molecules, and counterions (Na^+^ and Cl^−^) without performing system equilibration.

### Electrophysiology

For acute brain slicing, 5-week-old C57BL/6 J mice were anesthetized with isoflurane and decapitated. Transverse slices of the hippocampus (300 μm) were prepared in ice-cold NMDG-based cutting solutions (93 mM NMDG; 2.5 mM KCl; 1.2 mM NaH2PO4; 30 mM NaHCO3; 20 mM HEPES; 25 mM glucose; 5 mM sodium ascorbate, 2 mM thiourea, 3 mM sodium pyruvate, 10 mM MgCl2, 0.5 mM CaCl2, pH adjusted to 7.3 with HCl, 310 mOsm) on the D.S.K Linear Slicer pro7 (Dosaka EM Co., Ltd) and recovered for 15 min at 32 °C in the same solution. Slices were rerecovered in oxygenated ACFS solution (126 mM NaCl; 24 mM NaHCO3; 2.5 mM KCl; 1 mM NaH2PO4; 2 mM MgCl2; 10 mM glucose). Spontaneous IPSCs were recorded under oxygenated ACFS solution conditions by a whole-cell voltage clamp. Recording electrodes (6–8 MΩ) fabricated from standard wall borosilicate glass (GC150F-10, Warner Instrument Corp., USA) were filled with a CsCl-based internal solution (135 mM CsCl; 4 mM NaCl; 0.5 mM CaCl2; 10 mM HEPES; 5 mM EGTA, 0.5 mM Na2-GTP; 2 mM Mg-ATP; 1 mM QX-314). For the acute PD treatment and sIPSC recordings, ACSF containing 10 µM PD was applied after establishment of the baseline. For long-term PD treatment and sIPSC recording, slices were incubated in ACSF containing PD (0, 0.3, 1, 3, 10, 30 μM) for at least 1 h, and whole-cell voltage-clamp recordings were performed in the same solutions. sIPSCs were recorded for at least 5 min. For sIPSC frequency and amplitude analysis, Mini Analysis Program software (Synaptosoft) was used. Data obtained with a holding current under −300 pA at baseline were excluded from the analysis.

### Statistical analysis

The data in this study are representative of two or three independent experiments with triplicate samples and are presented as the mean ± SEM. Statistical analysis was performed using Student’s *t*-test or one-way ANOVA followed by Tukey’s post hoc test. A *p* value less than 0.05 was considered to indicate statistical significance (*P < 0.05; **P < 0.01; ***P < 0.001; ****P < 0.0001; NS not significant). For all statistical analyses, Prism v.9.0.0 was used (GraphPad Software).

## Supplementary information

Supplementary Information
