# Supplementary material for: Platycodin D, a natural component of Platycodon grandiflorum, prevents both lysosome- and TMPRSS2-driven SARS-CoV-2 infection by hindering membrane fusion
Source: Exp Mol Med. 2021 May 25;53(5):956–72. doi: 10.1038/s12276-021-00624-9 (PMC8143993; doi:10.1038/s12276-021-00624-9)
Supplement: Supplementary file 1 — Supplementary Information [file 12276_2021_624_MOESM1_ESM.docx]

**Supplementary information, Fig. S1 Transduction efficiency of pSARS-CoV-2.** Parental H1299 cells and H1299 cells expressing ACE2 were transduced with pSARS-CoV-2 containing firefly luciferase gene. At 2-day post-transduction, transduction efficiency was determined by measuring luciferase activity in cell lysates. The activity obtained from untransduced cells were used for normalization.


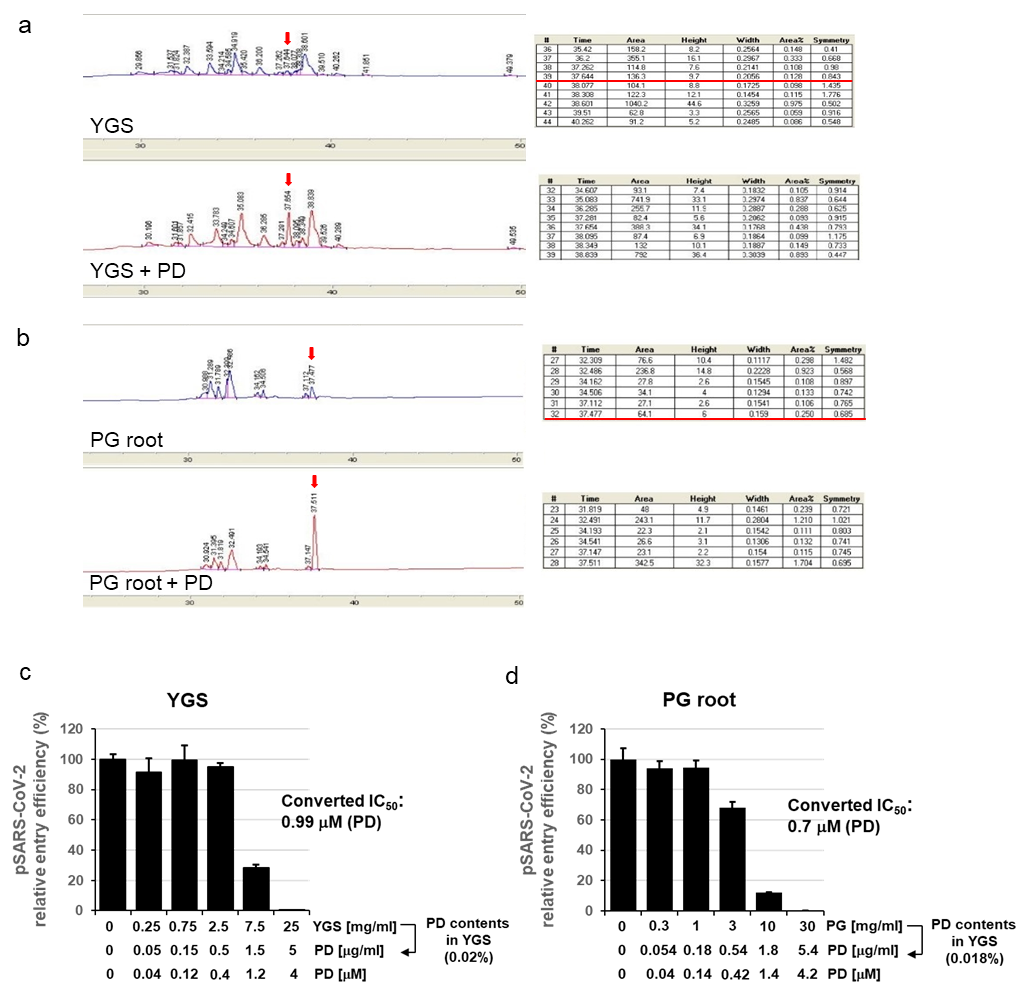


**Supplementary information, Fig. S2 PD is the main compound that exhibits anti-SARS-CoV-2 activity in YGS and PG root. a** Comparison of HPLC profiles for two samples of YGS (750 mg) and YGS (750mg) plus PD (0.25 mg) indicates that #39 (red arrow) is a peak for PD. The peak area for PD (red line in the table) is 136.3. **b** HPLC profiles of PG root (660 mg) and PG root (660 mg) plus PD (0.25 mg) indicates that #32 is a peak for PD and its value is 64.1. When these area value are put into the regression equation of “Y=2356*X-220.1” (R^2^ is 0.9989), which is obtained by a quantitative HPLC analysis using external standard calibration method (**Supplementary information, Fig. S3**), PD concentration can be calculated as 0.1513 mg/ml of PD in 750 mg of YGS (which is equivalent to 0.020%) and 0.1206 mg of PD in 660 mg of PG root (which is equivalent to 0.018%). **c, d** pSARS-CoV-2 entry assay using ACE2^+^ with serial three-fold dilutions of the stock solution of YGS and PG root. After converting mg/ml of YGS and PG root to mM of PD, the corrected IC_50_ of PD was 0.99 μM and 0.7 μM, respectively, which were very similar to IC_50_ of 0.69 μM obtained from the pSARS-CoV-2 entry assay with PD (**Fig. 1d**), indicating that PD is the main compound that exhibits anti-SARS-CoV-2 activity in the YGS powder and PG root.


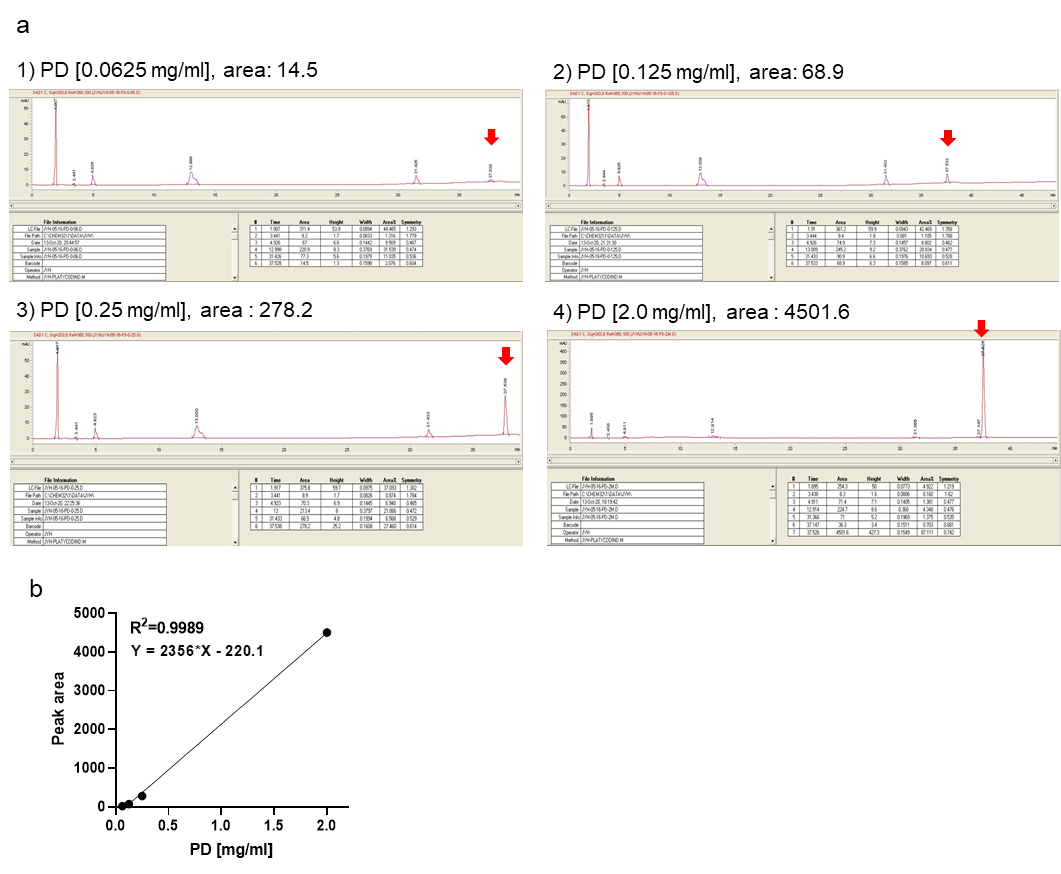


**Supplementary information, Fig. S3 Quantification of PD by HPLC using external standard calibration method. a, b** HPLC quantitative analysis using external standard method. Calibration curve (**b**) was created with values of the peak area for 0.0625, 0.125, 0.25, and 2.0 mg/ml of PD (**a**). R2 value and regression equation was obtained as 0.9989 and “Y=2356*X-220.1”, respectively (**b**).


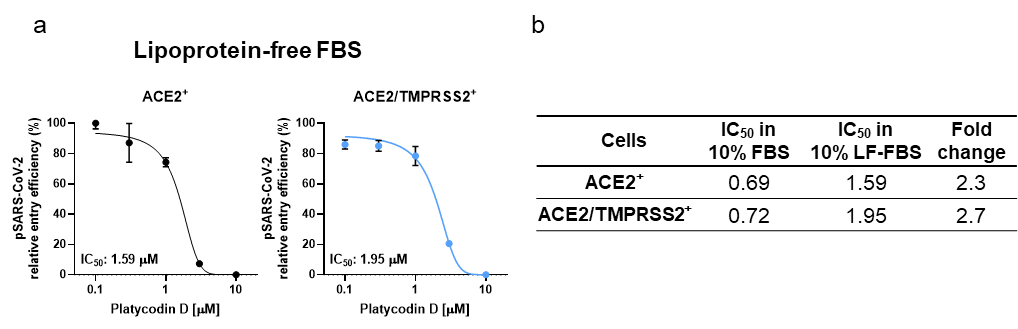
.

**Supplementary information, Fig. S4 PD’s inhibitory action against SARS-CoV-2-entry is cholesterol-dependent. a** ACE2^+^ and ACE2/TMPRSS2^+^ cultured in 10% lipoprotein-fee FBS (LF-FBS)-containing media for 24 h were pre-treated with indicated concentrations of PD for 1 h and further transduced with pSARS-CoV-2 in 10% LF-FBS-containing media in the presence of PD. After culture for 24 h, transduction efficiency was quantified by measuring the activity of firefly luciferase in cell lysates. Data are representative of two independent experiments with triplicate samples. Error bars indicate SEM. **b** Comparison of IC_50_ of PD between in normal culture condition (**Fig. 1d, l**) and in lipoprotein-free culture condition.
